# Trends of the Global, Regional, and National Incidence of Measles, Vaccine Coverage, and Risk Factors in 204 Countries From 1990 to 2019

**DOI:** 10.3389/fmed.2021.798031

**Published:** 2022-01-20

**Authors:** Ruitong Wang, Wenzhan Jing, Min Liu, Jue Liu

**Affiliations:** ^1^Department of Epidemiology and Biostatistics, School of Public Health, Peking University, Beijing, China; ^2^National Health Commission Key Laboratory of Reproductive Health, Peking University Health Science Center, Beijing, China; ^3^Institute for Global Health and Development, Peking University, Beijing, China

**Keywords:** measles, trend, incidence, vaccination, risk factor, prevention

## Abstract

**Background:**

Measles is a highly contagious disease that has caused global morbidity and mortality. Though great progress has been made in measles elimination, the resurgence of measles has been observed in recent years. As extant literature were mainly confined to data of local area, we conducted a systematic analysis to explore the trends of the incidence of measles, coverage rate, and the risk factors from 1990 to 2019 on global, regional, and national levels.

**Methods:**

Data on cases of incidence, age standardized rate (ASR), vaccine coverage, and risk factors of measles were retrieved from the Global Burden of Disease (GBD) Study 2019 database. Estimated averaged percentage change (EAPC) of ASR was calculated to quantify the trends of measles incidence. Pearson correlation was applied to assess the association of EAPC and measles-containing vaccine coverage rate with socio-demographic index (SDI) in 2019, and the correlation between ASR and measles-containing vaccine coverage rate.

**Results:**

Globally, there was a significant decrease in the number (84.18%) and ASR (6.13%, 95% CI: 5.41–6.84%) of measles incidence from 1990 to 2019. More than 80% of incidence cases were attributed to children under 5 and the proportion was highest in low SDI region. Countries and territories with low ASRs are mostly clustered in North America, Southern Latin America, and Western Europe, whereas those with high ASRs are mainly clustered in Africa, East Asia, and South Asia. Lower SDI was associated with higher ASR and lower vaccine coverage rate. The more the SDI value was further away from 0.6, the smaller the absolute value did EAPC have. Child wasting accounted for the most measles-related death cases, followed by child underweight, child stunting, and vitamin A deficiency.

**Conclusions:**

Measles eradication is feasible, but urgently demands political commitment, financial support, and public engagement. In the context of COVID-19 pandemic and the global resurgence of measles, surveillance systems and routine immunization programs should be improved, while vaccine hesitancy needs to be addressed.

## Introduction

Measles is a highly contagious, acute febrile disease caused by the measles virus. It is transmitted by respiratory route through respiratory droplets or aerosolized particles (1). Measles infection is usually characterized by an erythematous maculopapular rash, and complicated with damage in multiple organs including pneumonia, diarrhea, and CNS disorder (2). Measles is one of the most contagious diseases, with the basic reproduction number (R0) estimate varying more than 12–18, which is far higher than that of influenza virus (R0 = 2–3) (3), ancestral SARS-CoV-2 virus (R0 = 2–4) (4), and the Delta variant of SARS-CoV-2 virus (R0 = 5–8) (5).

Measles is a vaccine-preventable disease, as lifelong immunity follows vaccination. Subpopulations that have not received measles vaccination or constantly miss out on vaccines (particularly travelers) could trigger localized measles outbreaks (2). Unvaccinated young children are at the highest risk of measles and its severe complications (6). With the implementation of routine vaccination programs, the age distribution of measles shifts from children under 5 to older children and adults, including pregnant women (7). Poor nutritional status, particularly vitamin A deficiency, could be risk factors for measles (7).

As a leading cause of global morbidity and mortality before the introduction of vaccines, measles was responsible for more than 2 million cases of deaths annually (2). With the increasing vaccine coverage rate of measles, the annual reported measles incidence and deaths decreased by 88% and 84% from 2000 to 2016 (8). However, due to the suboptimal vaccination campaigns and policy initiatives in many countries, global resurgence in measles has been observed since 2016 (9). By the end of 2019, no WHO region (African region, Eastern Mediterranean region, European region, region of the Americas, South-East Asia region and Western Pacific region) had achieved and maintained measles elimination. In 2019, African region had the highest measles incidence (567 cases per 1 million population), followed by European region (116 cases per 1 million population) (8). This deteriorating situation may result in the reestablishment of endemic measles transmission in countries that have previously eliminated measles virus, which poses a threat to global health, utilizes substantial medical resources, and hinders the progress in the goal of measles eradication. The eradication of measles virus is of great necessity to promote global health and urgently needs joint efforts of the global health community and organizations.

Great progress has been made in reducing the incidence of measles. However, resurgence of measles has been observed in recent years. Studies on the global scale are urgently needed to understand the global landscape of measles, yet extant literature of incidence of measles were mainly confined to data of local area. Additionally, studies about the global disease burden and the trend of measles are lacking. Hence, we conducted an analysis based on data from Global Burden of Disease (GBD) Study 2019 to provide a greater emphasis on understanding the trends and risk factors of the incidence of measles in 204 countries from 1990 to 2019 on global, regional, and national levels. This study could provide a more comprehensive perspective on tailored strategies for measles prevention in targeted regions and countries, and provide implications for the global goal of measles eradication.

## Materials and Methods

### Data Source

Data on cases of incidence and age standardized incidence rate of measles in 204 countries and territories during 1990–2019 were retrieved from the Global Health Data Exchange (GHDx) query tool (10). The methods for the estimations of measles burden have been detailed elsewhere (11). Data on incidence are identified from systematic review of published studies, searches of government and international organization websites, published reports, and so on. DisMod-MR 2.1 is a Bayesian meta-regression tool that was used to model data and generate estimates of incidence. Four specific risk factors for measles mortality were defined and sought in the Global Burden of Diseases, Injuries, and Risk Factors Study 2019, which included child underweight, child wasting, child stunning, and vitamin A deficiency (12). The definitions of risk factors were clarified in the previous study (12).

Countries and territories were classified into 5 regions by socio-demographic index (SDI) (regions with a high, high-middle, middle, low-middle, or low SDI). Socio-demographic index is a composite indicator of income per capita, average years of schooling, and fertility rates that ranges from 0 to 1. Higher SDI value represents higher income, more years of schooling, and lower fertility rate. Statistics about SDI values of 204 countries and territories can be retrieved from GBD database.

Measles-containing vaccine coverage was defined as the proportion of children who received measles-containing vaccine, first dose (MCV1), and measles-containing vaccine, second dose (MCV2) through a routine immunization program (13). Data about MCV1 and MCV2 coverage rates in 204 countries from 1990 to 2019 are available in the GHDx website (http://ghdx.healthdata.org/gbd-results-tool).

### Statistical Analysis

The absolute number of measles incidence cases in 204 countries and territories and 5 SDI regions with 95% uncertainty intervals (UIs) were used. Due to the necessity of standardization when comparing several populations with different age structures or the same population with changing age structures over time, age-standardized incidence rate (ASR) of measles with 95% UIs were calculated by applying the age-specific rates for each location, sex, and year to a GBD World Standard Population, and were used to describe the epidemic status of measles. Change in measles incidence cases from 1990 to 2019 and estimated averaged percentage change (EAPC) of ASR with 95% confidence intervals (CIs) were calculated to quantify the trends of measles incidence from 1990 to 2019. Pearson correlation was applied to assess the association of EAPC and measles-containing vaccine coverage rate with SDI in 2019, and the correlation between ASR and MCV coverage rate from 1990 to 2019 at national levels, respectively. We used stacked barcharts to visualize risk factors that contributed to measles-related death from 1990 to 2019 in five SDI regions.

Relative changes of measles incidence cases from 1990 to 2019 was defined as $\frac{c1-c2}{c2}\times 100\%$, where c1 represents number of measles incidence cases in 2019 and c2 represents number of measles incidence cases in 1990. EAPC is widely used to measure the trend of ASR over time, which could serve as an indicator for the shifting patterns of disease among regions. EAPC of incidence was calculated by regression lines that were fitted to the natural logarithm of the ASR. The model was *y* = α + βx + ε, where *y* = ln(ASR), and *x* = calendar year. When ASR equals to 0, we replaced it with 0.01(14). EAPC was then calculated as 100 × (e^β^ – 1) and its 95% CI was also calculated. ASR was deemed increasing if the EAPC estimation and its 95% CIs were both > 0, and vice versa.

All statistical analysis and data visualizations were performed using R (Version 4.1.0). A *p* < 0.05 was considered significant.

## Results

### Global Trends in Incidence of Measles Infection

Globally, there was a consistent decrease in the number of measles incidence cases across the years, which decreased from 80,933,448.62 in 1990 to 12,806,077.45 in 2019 (Supplementary Figure 1). The ASR of measles infection decreased annually and reduced by 6.13% (95% CI 5.41–6.84%) per year from 1278.81 per 100,000 in 1990 to 191.04 per 100,000 in 2019 (Table 1). The ASR of measles infection markedly decreased from 1995 to 2015, whereas it slightly decreased from 1990 to 1995 and from 2015 to 2019 (Supplementary Figure 2).

**Table 1:** The number of incidence cases and age-standardized incidence rates (ASR, per 100,000) of measles in 1990 and 2019, and their temporal trends from 1990 to 2019.

**Characteristics**	**Number of incidence cases (per 1,000)**			**Age-standardized incidence rate (ASR, per 100,000)**		
	**1990 (90% UI)**	**2019 (95%UI)**	**Percentage change (%)**	**1990 (95%UI)**	**2019 (95%UI)**	**EAPC (95%CI)**
**Global**	80,933.45 (27,850.06–180,670.24)	12,806.08 (4,548.91–27,689.60)	−84.18	1,278.81 (440.07–2,854.65)	191.04 (67.85–413.04)	−6.13 (−6.84 to −5.41)
**Sex**						
Male	41,349.19 (14,226.07–92,295.77)	6,537.64 (2,324.96–14,150.89)	−84.19	1,269.16 (436.67–2,832.81)	189.02 (67.21–409.10)	−6.18 (−6.89 to −5.45)
Female	39,584.26 (13,623.99–88,374.48)	6,268.44 (2,223.95–13,538.70)	−84.16	1,288.87 (443.62–2,877.42)	193.18 (68.52–417.20)	−6.17 (−6.88 to −5.46)
**Socio-demographic index**							
Low	19,924.23 (6,706.60–44,759.83)	5,236.66 (1,818.74–11,625.77)	−73.72	2,096.11 (705.56–4,708.83)	307.32 (106.73–682.23)	−6.79 (−7.40 to −6.18)
Low-middle	30,116.25 (10,191.34–68,034.24)	3,345.02 (1,188.68–7,263.52)	−88.89	1,800.43 (609.27–4,067.29)	191.11 (67.91–415.02)	−6.44 (−7.51 to −5.36)
Middle	23,567.82 (8,137.57–51,880.50)	2,801.04 (983.77–5,896.72)	−88.11	1,146.5 (395.87–2,523.83)	149.82 (52.61–315.45)	−6.04 (−6.50 to −5.58)
High-middle	6,279.33 (2,222.34–13,664.54)	914,277.31 (424,826.33–1,782,654.69)	−85.44	596.42 (211.06–1,297.87)	108.78 (50.53–212.08)	−6.57 (−6.97 to −6.17)
High	1,008.86 (518.81–1,960.51)	62.56 (32.48–118.67)	−93.80	171.81 (88.28–334.03)	11.59 (6.02–21.98)	−11.21 (−12.81 to −9.58)
**GBD region**						
High-income Asia Pacific	6.83 (6.66–6.98)	1.82 (1.73–1.90)	−73.36	6.36 (6.21–6.51)	2.4 (2.29–2.51)	−13.16 (−18.35 to −7.64)
Central Asia	18.19 (17.93–18.46)	30.89 (30.54–31.25)	69.80	19.42 (19.14–19.71)	32.18 (31.82–32.56)	2.78 (−2.71 to 8.58)
East Asia	8,363.23 (2,807.02–18,210.78)	893.99 (302.10 −2,012.54)	−89.31	694.38 (233.06–1,512.02)	106.04 (35.83–238.7)	−9.78 (−11.24 to −8.29)
South Asia	33,015.11 (11,096.19–73,864.67)	3,249.11 (1,131.87–6,945.29)	−90.16	2,050.89 (689.3–4,588.55)	193.22 (67.31–413.07)	−5.98 (−7.37 to −4.56)
Southeast Asia	11,493.63 (3,917.29–25,897.47)	1,526.76 (523.61–3,299.41)	−86.72	1,921.74 (654.98–4,330.06)	274.33 (94.09–592.83)	−6.16 (−6.69 to −5.63)
Australasia	7.60 (7.43–7.79)	2.55 (2.45–2.65)	−66.46	48.45 (47.33–49.64)	13.76 (13.23–14.29)	−12.97 (−18.34 to −7.24)
Caribbean	10.70 (10.24–11.57)	0.37 (0.13–0.90)	−96.53	25.85 (24.74–27.94)	0.92 (0.33–2.24)	−9.18 (−10.77 to −7.55)
Central Europe	141.72 (141.04–142.46)	15.27 (15.04–15.51)	−89.22	155.06 (154.31–155.87)	26.3 (25.9–26.71)	−0.42 (−5.40 to 4.82)
Eastern Europe	28.92 (28.57–29.24)	116.21 (115.58–116.83)	301.82	16.65 (16.45–16.83)	92.37 (91.88–92.87)	7.01 (1.15 to 12.52)
Western Europe	234.03 (233.07–234.93)	12.86 (12.62–13.08)	−94.50	99.18 (98.77–99.56)	5.69 (5.58–5.78)	−13.00 (−16.52 to −9.32)
Andean Latin America	3.20 (3.09–3.31)	0.00431 (0.00051–0.00814)	−99.87	5.9 (5.68–6.1)	0.01 (0–0.01)	−21.60 (−29.13 to −35.95)
Central Latin America	110.35 (109.73–111.02)	0.86 (0.80–0.91)	−99.22	48.24 (47.97–48.53)	0.39 (0.36–0.41)	−23.74 (−33.54 to −12.48)
Southern Latin America	4.04 (3.91–4.16)	0.12 (0.10–0.14)	−97.06	7.79 (7.54–8.03)	0.24 (0.2–0.28)	−16.44 (−20.67 to −11.98)
Tropical Latin America	62.81 (62.34–63.28)	13.91 (13.67–14.16)	−77.85	35.22 (34.96–35.48)	8.43 (8.28–8.58)	−7.06 (−17.54 to 4.75)
North Africa and Middle East	8,948.67 (3,057.16–19,822.30)	1,066.26 (371.24–2,306.08)	−88.08	1,689.58 (577.26–3,742.48)	176.58 (61.48–381.88)	−9.53 (−10.33 to −8.72)
High-income North America	29.38 (28.86–30.14)	1.63 (1.48–1.88)	−94.45	13.49 (13.25–13.84)	0.76 (0.68–0.87)	−10.34 (−14.46 to −6.04)
Oceania	171.38 (58.71–378.88)	102.43 (39.28–220.60)	−40.23	1,756.13 (601.58–3,882.34)	559.44 (214.77–1,204.37)	−4.28 (−5.16 to −3.40)
Central Sub-Saharan Africa	2,261.59 (761.29–5,084.13)	993.62 (341.32–2,255.02)	−56.07	2,150.94 (724.05–4,835.48)	482.33 (165.69–1,094.66)	−7.23 (−7.92 to −6.53)
Eastern Sub-Saharan Africa	7,331.88 (2,488.96–16,572.50)	2,057.02 (720.79–4,627.85)	−71.94	2,069.1 (702.4–4,676.86)	321.95 (112.82–724.34)	−6.68 (−7.30 to −6.05)
Southern Sub-Saharan Africa	1,202.42 (418.90–2,671.59)	245.00 (83.97–540.37)	−79.62	1,673.93 (583.15–3,719.24)	299.87 (102.78–661.41)	−5.61 (−7.42 to −3.77)
Western Sub-Saharan Africa	7,487.76 (2,527.48–16,980.95)	2,475.40 (856.16–5,628.48)	−66.94	2,135.71 (720.93–4,843.82)	341.87 (118.25–777.32)	−6.99 (−7.80 to −6.18)

Among all the age groups, children aged under 5 accounted for the largest proportion of incidence cases from 1990 to 2019, with a minimum of over 80%. In 2019, an estimate of 10,959,898.81 measles incidence cases were attributed to children aged under 5, which accounted for 85.58% of the total incidence cases. The proportion had an overall decline from 1990 to 2017, and began to rise after 2017 (Supplementary Figure 3). No apparent differences of number of incidence cases and ASR by sex were observed (Table 1).

### Incidence of Measles Infection in Five SDI Regions and 21 GBD Regions

The ASR of measles infection in five SDI regions exhibited distinct diversities (Table 1; Supplementary Figure 2). Lower SDI regions generally presented higher ASR. From 1990 to 2019, low SDI and low-middle SDI regions had the highest ASR, followed by middle SDI region, high-middle SDI region, whereas high SDI region had the lowest ASR. In 2019, the ASR of measles infection in high SDI region was 11.59 per 1,00,000, whereas the rate was 307.32 per 1,00,000 in low SDI region. Five SDI regions had an overall decreasing trend of ASR, among which high SDI regions had the largest decreasing trend (EAPC = −11.21, 95%CI: −12.81 to −9.58). However, ASR in high SDI regions continued to rise in the past 5 years.

**Figure 1:**
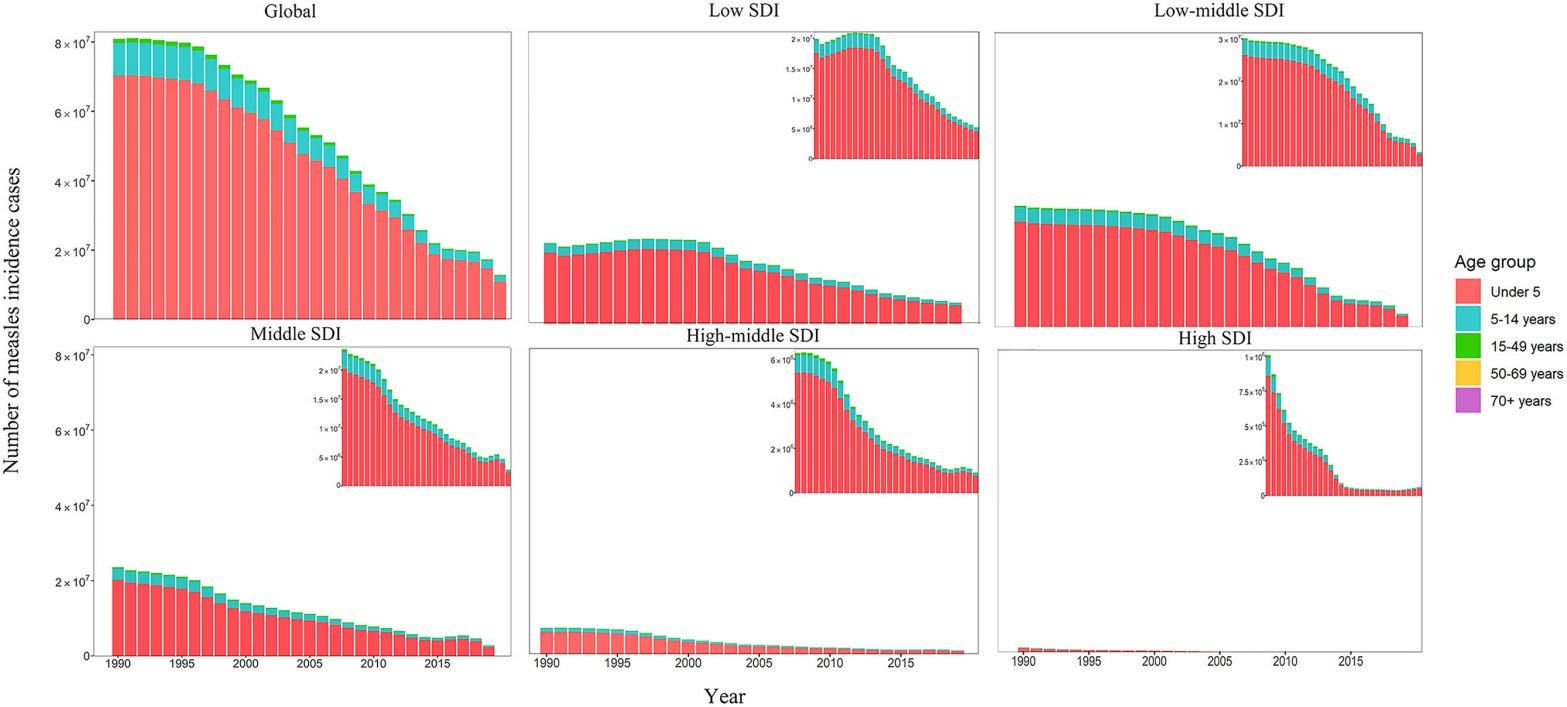
The number of measles incidence cases by age groups and SDI regions. SDI: socio-demographic index.

The distribution of number of measles cases in five SDI regions generally parallels that of ASR, and that of higher SDI regions generally presented lower numbers of measles cases (Supplementary Figure 1; Table 1). In 2019, low SDI region had the highest number of measles incidence cases (5,236,664.33). Cases in low SDI region and low-middle SDI region accounted for 69.43% of global measles incidence cases. High SDI region had the lowest number of measles cases (62,557.8 in 2019) and the largest percentage change (−93.80%). The number of measles cases in high SDI region had a sustained increase in recent 5 years. Children aged under 5 had the highest proportion of measles incidence cases in all five SDI regions, compared to other age groups (Figure 1). The proportion of children aged under 5 was highest in low SDI region. The decrease in measles incidence cases in five SDI regions were almost completely determined by the decrease among children aged under 5. In low-middle SDI and middle SDI regions, measles incidence cases among children aged under 5 decreased by 38.07 and 39.46%, respectively from 2018 to 2019, which were the highest decreasing rates from 1990 to 2019. Since 2015, measles incidence cases had increased by 54.79% in children aged under 5 in high SDI region.

In terms of 21 GBD regions (Table 1), Oceania had the highest ASR of measles incidence in 2019 (559.44 per 100,000 population), followed by Central, Western, and Eastern Sub-Saharan Africa (482.33, 341.87, 321.95 per 100,000 population, respectively). Central, Western, and Eastern Sub-Saharan Africa had the highest ASRs in 1990 as well, followed by South Asia. Andean Latin America, Southern Latin America, Central Latin America, high-income North America had the lowest ASRs in both 1990 (all below 10.00 per 100,000 population) and 2019 (all below 1.00 per 100,000 population). The number of measles incidence cases in Eastern Europe and Central Asia increased by 301.82 and 69.80%, and Eastern Europe had an increasing trend of ASR from 1990 to 2019 (EAPC = 7.01, 95%CI: 1.15–12.52). Andean Latin America had the largest percentage decrease in incidence cases (−99.87%) from 1990 to 2019, whereas Central Latin America had the largest decreasing trend of ASR (EAPC = −23.74, 95%CI: −33.54 to −12.48).

### Incidence of Measles Infection in 204 Countries and Territories

**Figure 2:**
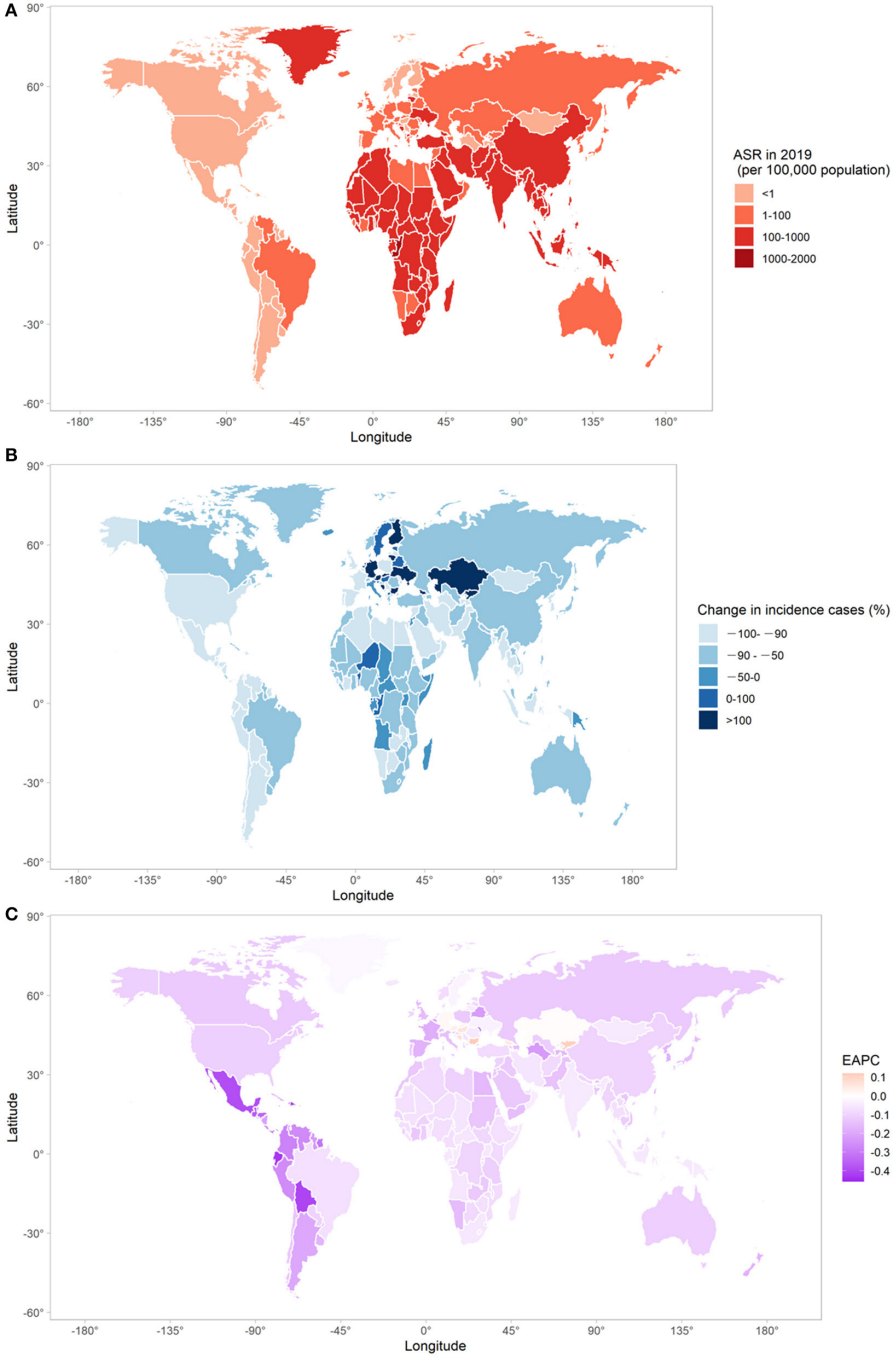
The global trends in measles incidence in 204 countries and territories. **(A)** The ASR of measles in 2019; **(B)** changes in number of measles incidence cases between 1990 and 2019; **(C)** the EAPC of measles ASR from 1990 to 2019. ASR, age-standardized rate; EAPC, estimated annual percentage change.

The ASR of measles, its EAPC, and change in measles incidence cases were heterogeneously distributed around the world (Figure 2). In 2019, there were overall 51 countries and territories with ASRs of below 1 per 100,000 population (Mexico, Peru, the United States of America, Canada, etc.), 63 countries with ASRs of 1 to 100 per 100,000 population (Japan, UK, Brazil, Italy, etc.), 81 countries with ASRs of 100 to 999 per 100,000 population (China, Saudi Arabia, India, Turkey, etc.), 8 countries with ASRs of 1,000 to 2,000 per 1,00,000 population (Congo, Bermuda, etc.), and only one country with ASR of over 2,000 per 100,000 population (Samoa, 2,630.65). Countries and territories with low ASRs (<1 per 100,000 population) are mostly clustered in North America, Southern Latin America, and Western Europe, whereas those with ASRs of 100 to 999 per 100,000 population are mainly clustered in Africa, East Asia, and South Asia. In 2019, India had the largest measles incidence cases (2,538,603.00), followed by China (874,501.20) and Nigeria (763,564.70), which accounted for 19.84%, 6.83%, and 5.97% of global measles cases.

Twenty nine out of 204 countries and territories experienced an increase in measles incidence cases in 2019 compared with 1990. Among them, there were Kazakhstan (63.60 folds), Bulgaria (13.07 folds), Slovakia (11.81 folds), Israel (7.55 folds), Finland (4.25 folds), Netherland (4.11 folds), and Germany (1.39 folds). Measles incidence cases in 30 countries and territories decreased by more than 99.00% (Mexico, Serbia, Chile, Jamaica, etc.). Seven countries and territories had an increasing trend of measles ASR from 1990 to 2019. Among them, Bulgaria had the largest increasing trend (EAPC = 0.12, 95%CI: 0.01–0.23), followed by Kyrgyzstan (EAPC = 0.11, 95%CI: 0.01–0.22), Bosnia and Herzegovina (EAPC = 0.07, 95%CI: 0.01–0.14), and Hungary (EAPC = 0.06, 95%CI: 0.02–0.11). Notably, the ASRs of more than one-third of global countries and territories (74 out of 204) increased year by year from 2015 to 2019 (Japan, United Kingdom, Spain, Congo, China, etc.).

### The Correlation Between EAPC and SDI

**Figure 3:**
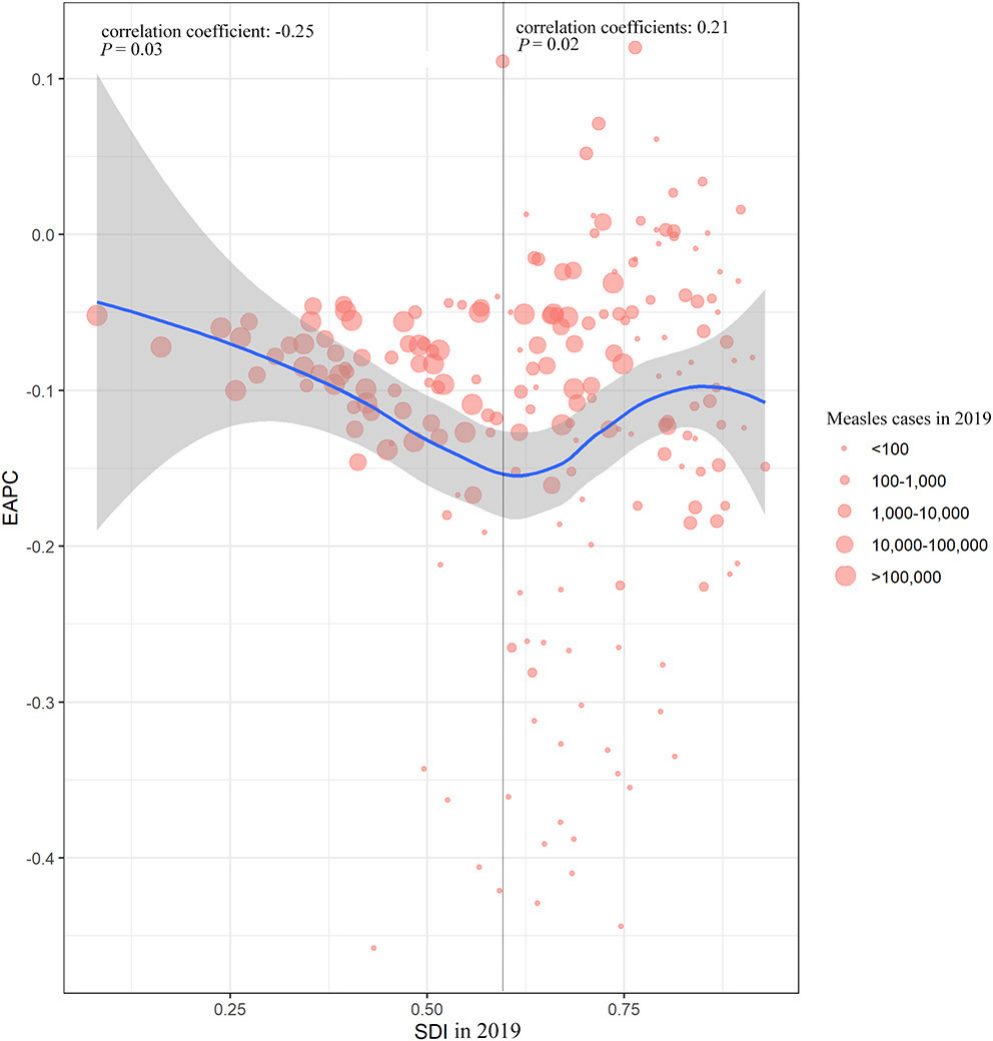
The correlation between measles EAPC and SDI in 2019 at national level. EAPC, estimated annual percentage change; SDI, socio-demographic index.

For countries and territories with SDI below 0.60 in 2019, a negative correlation was observed between EAPC and SDI (correlation coefficient = –0.25, *p* = 0.03). However, a positive correlation was found for SDI that is over 0.60 (correlation coefficient = 0.21, *p* = 0.02), indicating that countries with higher SDI in 2019 had a slower decreasing trend of measles ASR (Figure 3).

### Measles-Containing Vaccine Coverage Rate in 2019 and Its Correlation With SDI and ASR

**Figure 4:**
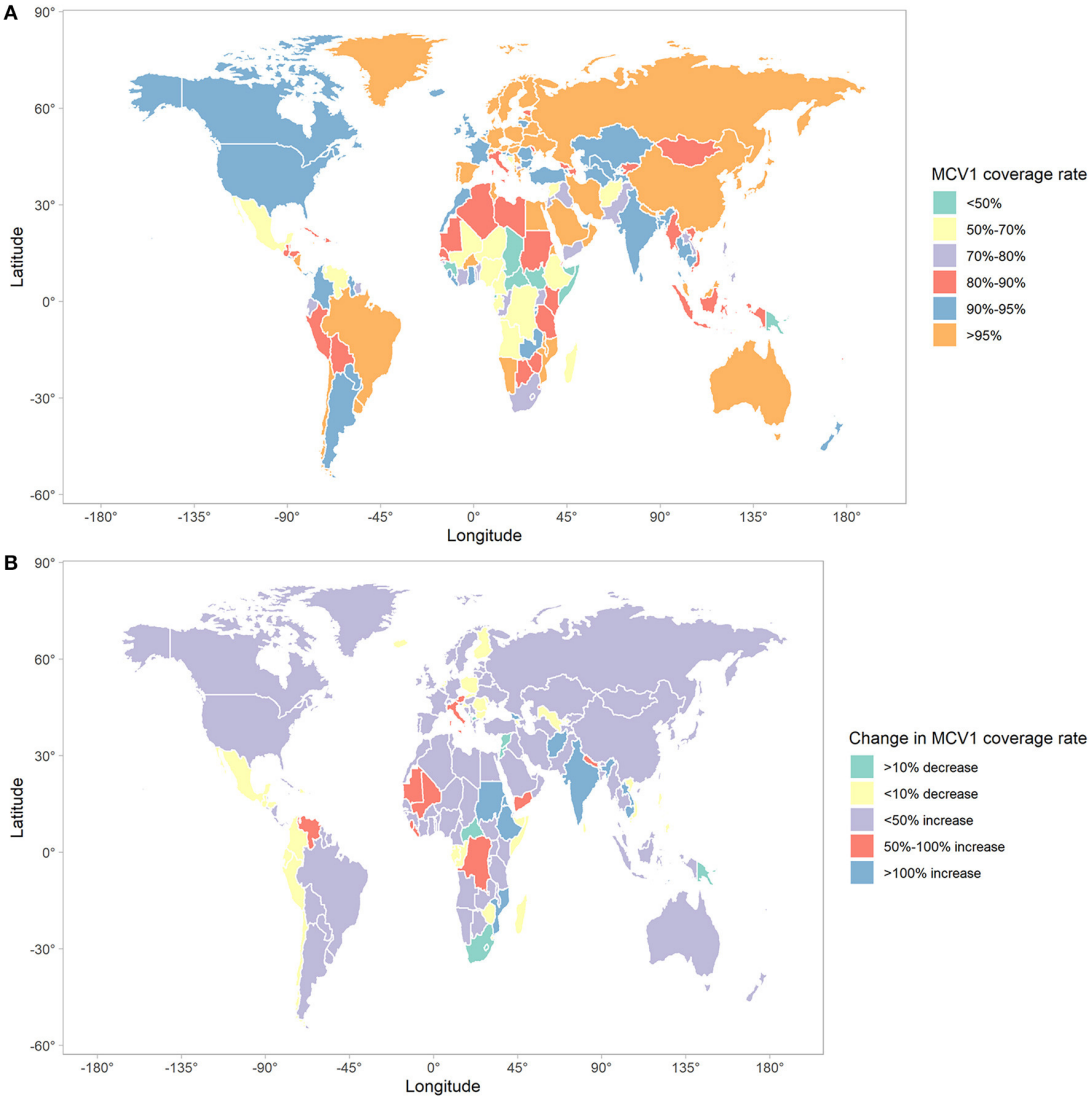
The global trends in MCV1 coverage in 204 countries and territories. **(A)** MCV1 coverage rate in 2019; **(B)** changes in MCV1 coverage rate between 1990 and 2019. MCV1, measles-containing vaccine, dose 1.

**Figure 5:**
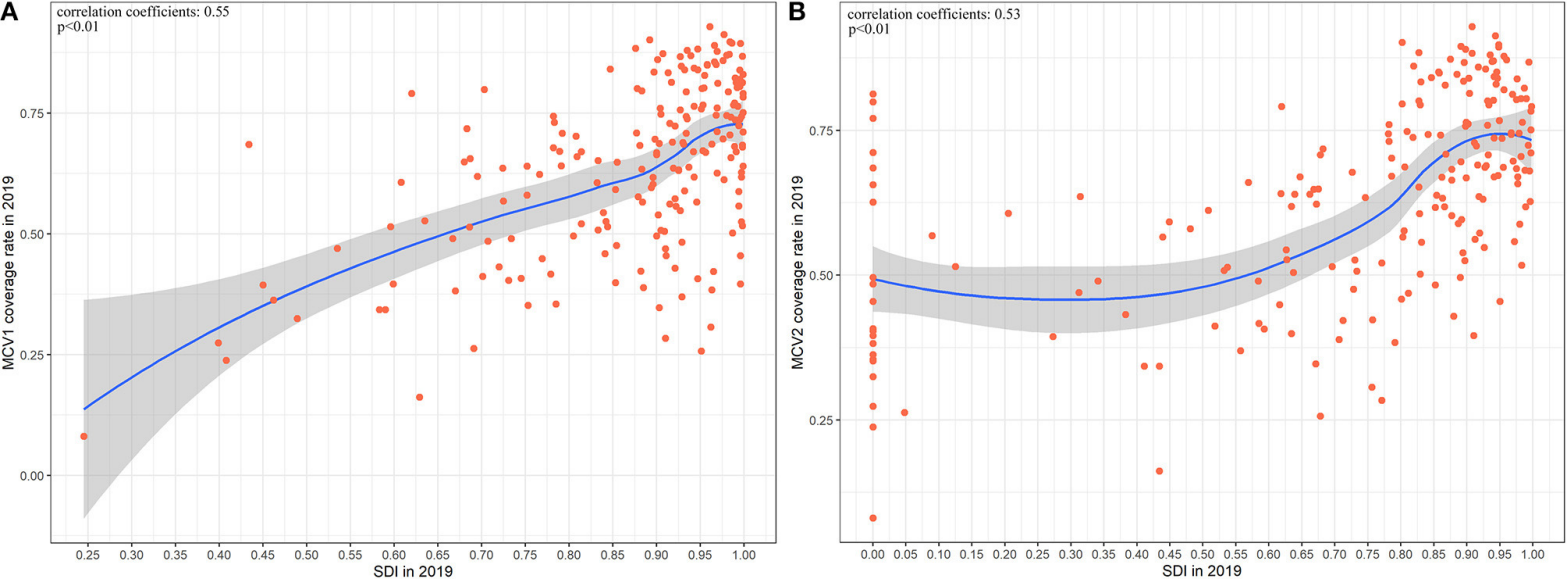
The correlation between measles-containing vaccine coverage and SDI in 2019 at national level. **(A)** The correlation between MCV1 coverage rate and SDI in 2019 at national level. **(B)** The correlation between MCV2 coverage rate and SDI in 2019 at national level. MCV1, measles-containing vaccine, dose 1; MCV2, measles-containing vaccine, dose 2; SDI, socio-demographic index.

MCV1 and MCV2 coverage rate and the corresponding change from 1990 and 2019 are shown in Figure 4; Supplementary Figure 4. In 2019, global MCV1 and MCV2 coverage rate was 83.6% (95%UI: 82.3–84.8%) and 68·1% (95%UI: 66.5–69.5). Out of 204 countries and territories, 74 reached the recommended MCV1 coverage rate of 95% (e.g., Brazil, Hungary, Australia, Russia, and Egypt), while MCV1 coverage rate was below 50% in 7 countries and territories (e.g., Guinea, Chad, Central African Republic). MCV2 coverage rate in 37 countries and territories was below 50%, 22 of which was 0. Only 36 countries reported the rate of over 95%. A decrease in MCV1 and MCV2 coverage rate from 1990 to 2019 was observed in 41 and 11 countries and territories, respectively. Among these, 10 countries and territories (Armenia, Croatia, Slovakia, Netherland, Iceland, Uzbekistan, Bulgaria, Czechia, North Macedonia, and Romania) experienced a decrease in both MCV1 and MCV2 coverage rate. Negative correlations were found between measles ASR and measles-containing vaccine coverage rate in 204 countries and territories throughout past 30 years (Supplementary Figure 5). SDI was positively associated with both MCV1 (correlation coefficients = 0.55, *p* < 0.01) and MCV2 coverage rate (correlation coefficients = 0.53, *p* < 0.01) in 2019 (Figure 5).

### Risk Factors for Measles-Related Death

**Figure 6:**
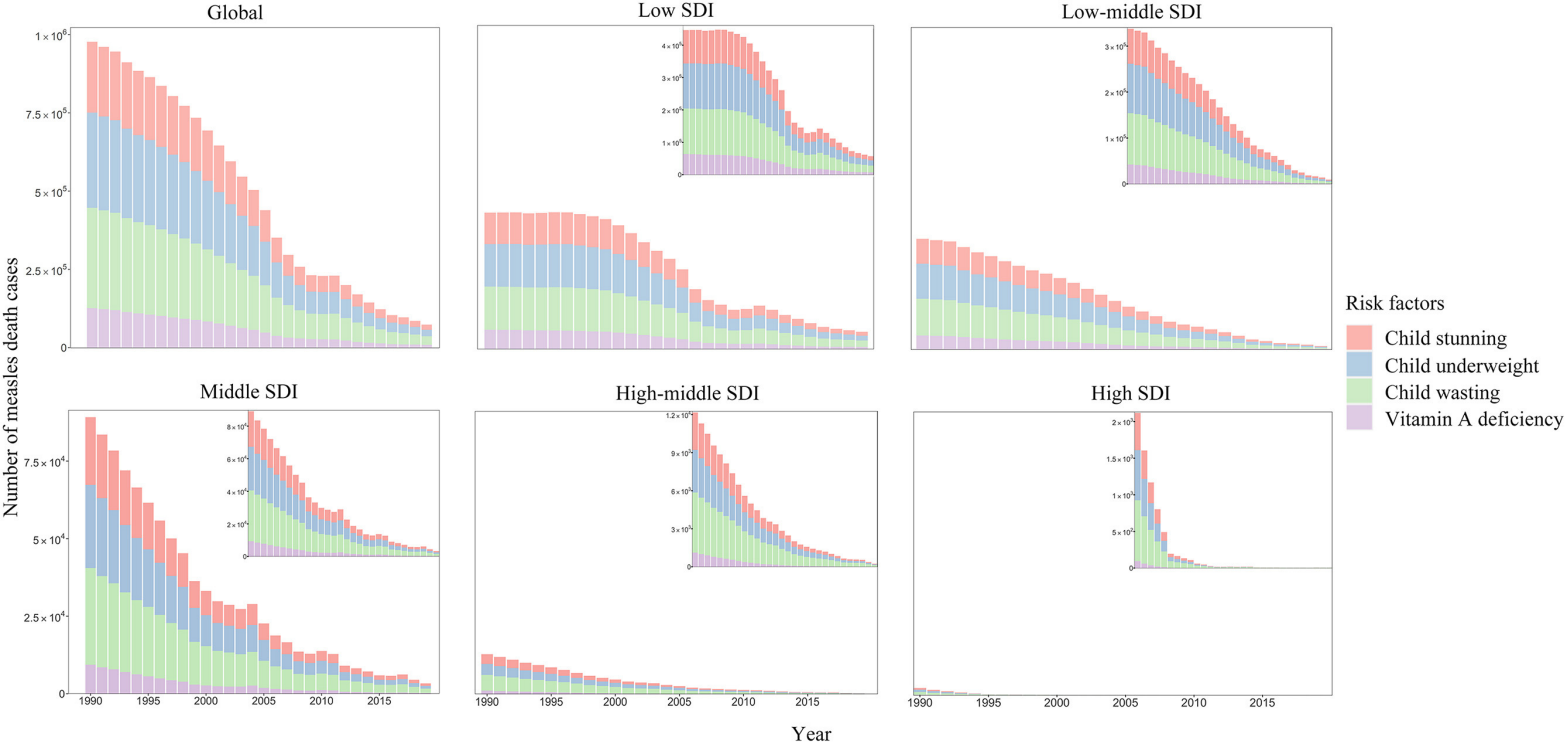
The number of measles death cases by risk factors and SDI regions. SDI, socio-demographic index.

According to GBD database, measles-related death cases can mainly be attributed to four risk factors from the most to the least: child wasting, child underweight, child stunting, and vitamin A deficiency (Figure 6). The proportion of death caused by child wasting increased steadily from 1990 to 2019, while the proportion of death due to the other three risk factors decreased slightly in the globe and in five SDI regions. In the high and high-middle SDI regions, child wasting is responsible for more than 50% of measles-related death in recent 5 years. In 2019, child wasting contributed to 61.51% of measles-related death in high SDI region, whereas the proportion was only 36.65% in the low SDI region. The proportion of vitamin A deficiency in high SDI region in 2019 (1.20%) was lower than that in the low SDI region (12.76%).

## Discussion

To our best knowledge, this is the first study to report global, regional, and national data on incidence of measles, vaccine coverage, and risk factors from 1990 to 2019, and to assess the epidemiological trend of measles incidence on a global scale based on GBD database. Globally, the ASR and number of measles cases decreased annually from 1990 to 2019 at a relatively rapid speed. On a regional level, lower SDI regions generally presented higher ASR and number of measles cases. Notably, a steady increase in ASR and number of measles cases in high SDI region was observed from 2015 to 2019. Majority of number of measles cases were composed of children aged under 5. The proportion of children aged under 5 among all the age groups was highest in low SDI region, and increased in high SDI region from 2015 to 2019. Countries with high ASR generally clustered in sub-Saharan Africa and Asia. It is noteworthy that Eastern Europe had an increasing trend of ASR. Despite significant decrease in global measles cases and ASR, the universal goal of measles eradication has not been achieved. Concerted efforts from global organizations and governments are needed to protect against measles resurgence and eventually achieve the goal of measles elimination and eradication.

Measles virus is a non-segmented, negative-sense RNA virus that remains antigenically monotypic (15). It is transmitted by respiratory droplets and small particle aerosols that could be facilitated by cough. Humans are the only hosts for measles virus and there are no known animal reservoirs that maintain its circulation (7). Additionally, measles can be readily diagnosed through the typical clinical syndrome that includes fever and rash, and identified with the support of potential traveling history to endemic areas (16). These virological and epidemiological features contribute to the possibility of measles eradication.

To date, there is still no specific antiviral therapies for measles (16). Another major obstacle to combat measles is its high contagiousness (3). Thus, the absence of specific therapies and high contagiousness underlines the necessity of prevention. Measles vaccination is the most effective method to prevent high-risk people (especially children aged under 5) from disease and control the spread of measles virus. Estimates suggest that measles vaccines have prevented over 21 million cases of death globally from measles virus infection since 2000 (17). Measles-containing vaccination has been recommended by the WHO since 1974, and has been introduced to the routine childhood vaccination schedule of many countries around the world to diminish children morbidity and mortality (18). Lifelong immunity to all circulating strains of measles virus is considered to be acquired after vaccination for the neutralizing the epitopes on the haemagglutinin protein of measles virus that bind with neutralizing IgG antibodies are highly conserved (17). In our work, we found that measles-containing vaccine coverage rate is negatively associated with ASR of measles in 204 countries and territories from 1990 to 2019, indicating the important role of measles vaccines in measles prevention and elimination. It is estimated that levels of population immunity as high as 89–94% are required to establish herd immunity and achieve measles elimination (2), which places higher demands on the uptake and coverage of vaccines, and urgently requires the implementation of global vaccination campaigns.

High vaccine coverage rate has always been required to proceed to the elimination and eradication of measles. In our work, we found that only 74 out of 204 countries and territories reached the recommended MCV1 coverage rate of 95%, 41 countries experienced a decrease in MCV1 coverage rate from 1990 to 2019. Another concern is that, due to the worldwide outbreak of COVID-19 pandemic, mass vaccination campaigns were advised to be temporarily suspended to prevent from community transmission of COVID-19 (19). The suspension in vaccination programs could largely affect measles vaccine coverage rate around the globe, especially in rural areas with insufficient health service resources and vaccine supply. Such concerns can be corroborated by secondary measles outbreaks during Ebola epidemic in Africa. Studies have shown that Ebola outbreaks in 2013–2016 triggered secondary measles outbreaks, for the interruption of vaccination campaigns and healthcare systems (20). In addition, as policy makers take social-distancing and lockdowns measures to curb the spread of COVID-19, it becomes difficult for people to approach health services or receive vaccination (21). For fear of contracting COVID-19 infection in densely populated areas, parents may delay routine childhood vaccination for their children (22). The number of MCV-1 and MCV-2 doses showed a reduction of 20.9 and 21.2% during COVID-19 lockdown in Sindh, Pakistan (22). Measles-containing vaccine administrations in America experienced an immediate decline the week after the national emergency declaration of COVID-19 pandemic (23). In England, measles-mumps-rubella vaccination counts were 19.8% lower in the 3 weeks after introduction of physical distancing measures than the same period in 2019 (24). Though COVID-19 protective measures (e.g., social distancing, lockdown, and wearing face masks) could prevent airborne transmission of measles virus, there could be potential measles outbreaks due to the suboptimal immunization coverage when social distancing policies become lenient or people do not comply with the protective measures. Because of the suspension of routine immunization programs, it is expected that 80 million children in 68 countries are at risk of developing vaccine-preventable disease according to the WHO (25). These situations could increase the risk of measles infection, and lead to severe morbidity and mortality in areas with poor vaccination coverage rate. Countries with high ASR and low vaccine coverage rate were grouped together in Sub-Saharan Africa. A benefit-risk analysis suggested that the benefit of sustaining routine childhood immunization in Africa far outweighs the risk of COVID-19 deaths during hospital visits (26). Policymakers should fully assess the risk of COVID-19 and benefits of routine immunization, and make flexible strategies to sustain immunization programs while controlling the epidemic situation of COVID-19. Numbers of global incidence cases of measles after the outbreak of COVID-19 awaits to be estimated in order to assess the impact of COVID-19 pandemic on measles transmission and infection.

Lower SDI was found to be associated with higher measles ASR and lower vaccine coverage rate in our study. This association could possibly be attributed to the following factors: high level of vaccine hesitancy, insufficient funding to support routine vaccination programs, and supply disruptions in measles vaccines (27). Some low SDI countries suffer from political conflicts and instability, which also hinders the implementation of vaccination programs. It is estimated that more than 99% of measles cases and deaths occur in low- and middle-income countries (21), which supports the findings of ours. Our result also showed that the more SDI value was further away from 0.6, the slower decreasing trends of ASR did countries have. For SDI below 0.6, countries with lower SDI had slower decreasing trend of ASR. This may be explained by the following reasons: (1) Countries with lower SDI generally lack high-quality medical resources and may be faced with multiple public health issues. Therefore, measles may be underappreciated and not adequately addressed for the shortage of vaccine supply or vaccination programs. (2) Countries with lower SDI broadly had higher baseline ASR. In this regard, the variation in ASR may be less significant. For SDIs that are over 0.6, countries with higher SDI had slower decreasing trend of ASR. This may be due to better surveillance and diagnosis systems in higher SDI countries.

Child growth failure and malnutrition were great threats to children's health (12). An analysis conducted by Caulfield et al. in 2004 found that 44.8% of measles-related death in young children were attributable to undernutrition, and estimated that 250,000 cases of measles deaths could be prevented by eradication of child undernutrition (28). Vitamin A deficiency is a major public health issue in low- and middle- income countries. It has long been reported to be associated with severe cases of measles in children in developing countries (29). Previous literature suggests that vitamin A supplement is an effective intervention to prevent measles mortality in children (30). The WHO also recommends vitamin A treatment of measles consisting of two doses of 50,000 IU for infants <6 months of age, 100,000 IU for those 6 months to 1 year of age, and 200,000 IU for individuals >1 year of age (31). Though the most recent Cochrane review suggested that vitamin A supplementation had no significant effect on measles-related mortality in children under 5, it also suggested that vitamin A supplementation reduced the incidence of measles (32). It is of great necessity for low and middle SDI countries (especially in areas with vitamin A deficiency) to implement vitamin A supplementation programs to improve vitamin A intake and reduce under-five incidence. Vitamin A supplementation should be implemented along with other strategies including dietary diversity and food fortification to prevent children growth failure (33).

Children aged under 5 have always been the highest population of measles incidence cases from 1990 to 2019. The increase in measles vaccine coverage and the decrease in contact between infectious individuals and populations at risk led to a shift toward older children and adults in age distribution in recent years (2), which is consistent with the findings of ours. We also found that the proportion of children aged under 5 was highest in low SDI region, compared to other SDI regions. This was possibly a consequence of low measles vaccine coverage rate and low levels of population immunity in densely populated area. To some extent, the upward shift in age distribution alleviates the burden of measles-related mortality, as morality rate was lower in older populations (34). However, more attention should be attached to the prevention of measles at the same time, as teenagers and adults have greater possibilities of traveling and may introduce virus to other regions and lead to potential outbreaks.

As discussed above, measles eradication is biologically feasible because of the virological and epidemiological features of measles. Owing to the high contagiousness and seriousness of measles, the eradication of measles has been on the global health agenda. As early as 2010, the WHO Strategic Advisory Group of Experts on Immunization stated that a goal for measles eradication should be established (35). In 2012, the Global Vaccine Action Plan set a goal for five out of six WHO regions to achieve measles elimination by 2020 (36). However, the goal of measles elimination was not reached and remained on the horizon. To make matters worse, global resurgence of measles has been observed in recent years. Take the Americas Region as an example. Measles was declared eliminated in the Americas Region in 2016. However, many countries including Brazil, Canada, Mexico, USA, and Venezuela reported measles cases during 2017 and 2018 (37). Measles resurgence also happened in other countries that were once free of endemic measles such as UK, Greece, and Mongolia (1). We also found an increasing trend of ASR in Eastern Europe. The resurgence of measles results from comprehensive factors. Apart from reasons such as suboptimal vaccine coverage rate and insufficient political commitment, global tourism is also an important source of virus introduction. Especially in recent years, millions of refugees traveled to and settled in Europe, which lead to high risk of measles transmission and may have spread measles to unvaccinated or undervaccinated populations. Global tourism and worldwide migration posed great burden for the elimination of measles (38).

In the context of COVID-19 pandemic and the global resurgence of measles, concerted efforts should be made to prevent global measles situation from lagging far behind the goal. In this regard, the focus of future work on measles eradication includes but are not limited to: (1) The standard of surveillance systems should be improved to ensure rapid and timely detection of measles cases, thus allowing a prompt contact tracking and could prevent further transmission. (2) Vaccine hesitancy toward measles needs to be addressed to raise the uptake of vaccines. Health care authorities and community workers should launch tailored campaigns about measles vaccination to inform people (especially parents) of the severity and high contagiousness of measles, the lifelong benefits of measles vaccination, and the safety and effectiveness of measles vaccines. The role of physicians in recommending measles-containing vaccines to parents should be underscored (39). (3) It's high time that routine immunization programs should be maintained and strengthened. Unvaccinated and undervaccinated children should be approached. Appropriate and context-specific strategies on maintaining routine immunization in the era of COVID-19 await to be rapidly developed to mitigate the adverse impact of COVID-19 on child immunization.

Measles eradication is a long-term cause that urgently demands political commitment, financial support, and public engagement. The achievement would bring significant improvement in global health (especially under-five health) and release substantial medical resources for other public health programs. In response to global resurgence of measles, low SDI region should initiate policy to effectively address the burden of child malnutrition and strengthen routine childhood immunization. In high SDI region, surveillance standard awaits to be improved. More studies are warranted in the future to track the process toward measles eradication and shed light on the orientation of future efforts.

The limitations of our study are listed as follows. First, the GBD estimates of measles incidence are based upon imperfect reporting and models. Due to the limited standard of surveillance, the actual number of measles cases may be vastly underestimated. With the improvement in surveillance systems, estimates can be more accurate. Second, measles-containing vaccine coverage rate on regional level could not be obtained for lack of relevant data. Third, the study focused on the incidence of measles rather than the prevalence or mortality of measles. The overall burden of measles awaits to be more comprehensively analyzed in the future.

## Data Availability Statement

Publicly available datasets were analyzed in this study. This data can be found here: http://ghdx.healthdata.org/gbd-results-tool.

## Author Contributions

RW, WJ, and JL contributed to conception and design of the study. RW performed the statistical analysis and wrote the first draft of the manuscript. JL, WJ, and ML reviewed and edited the manuscript. All authors contributed to manuscript revision, read, and approved the submitted version.

## Funding

This study was funded by National Natural Science Foundation of China (Nos. 72122001 and 71934002), National Key Research and Development Project of China (Nos. 2020YFC0846300 and 2019YFC1710301), and National Science and Technology Key Projects on Prevention and Treatment of Major Infectious Disease of China (No. 2020ZX10001002).

## Conflict of Interest

The authors declare that the research was conducted in the absence of any commercial or financial relationships that could be construed as a potential conflict of interest.

## Publisher's Note

All claims expressed in this article are solely those of the authors and do not necessarily represent those of their affiliated organizations, or those of the publisher, the editors and the reviewers. Any product that may be evaluated in this article, or claim that may be made by its manufacturer, is not guaranteed or endorsed by the publisher.

## Abbreviations

GBD: global burden of disease
SDI: socio-demographic index
MCV1, measles-containing vaccine: first dose
MCV2, measles-containing vaccine: second dose
UI: uncertainty intervals
ASR: age-standardized incidence rate
EAPC: estimated annual percentage change
CI: confidence interval.
