# Supplementary material for: Trends of the Global, Regional, and National Incidence of Measles, Vaccine Coverage, and Risk Factors in 204 Countries From 1990 to 2019
Source: Front Med (Lausanne). 2022 Jan 20;8:798031. doi: 10.3389/fmed.2021.798031 (PMC8810814; doi:10.3389/fmed.2021.798031)
Supplement: Supplementary file 1 [file Data_Sheet_1.DOCX]

Supplementary Material

## Supplementary Figures


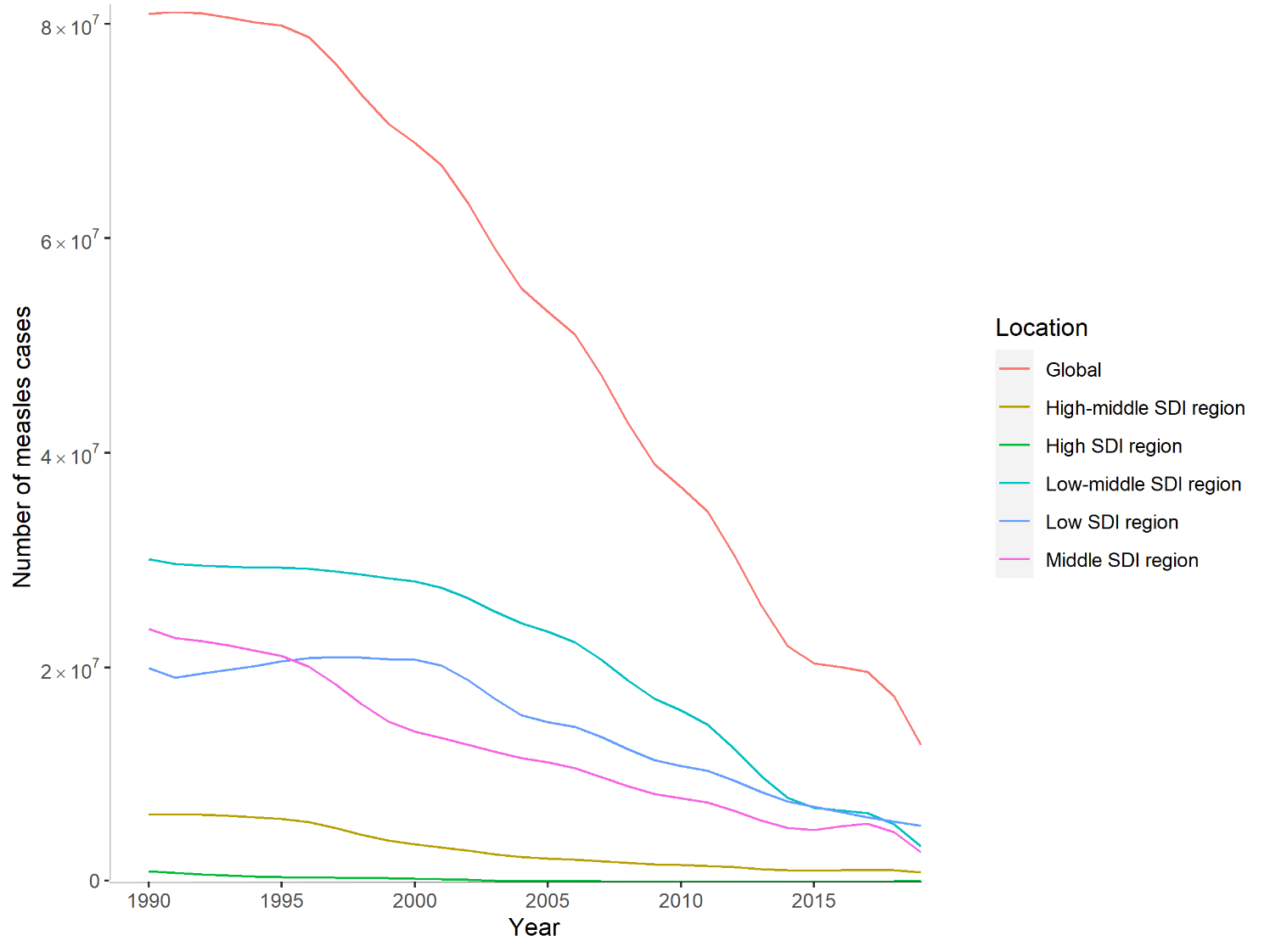


**Supplementary Figure 1.** The trends of number of measles cases by region from 1990 to 2019.


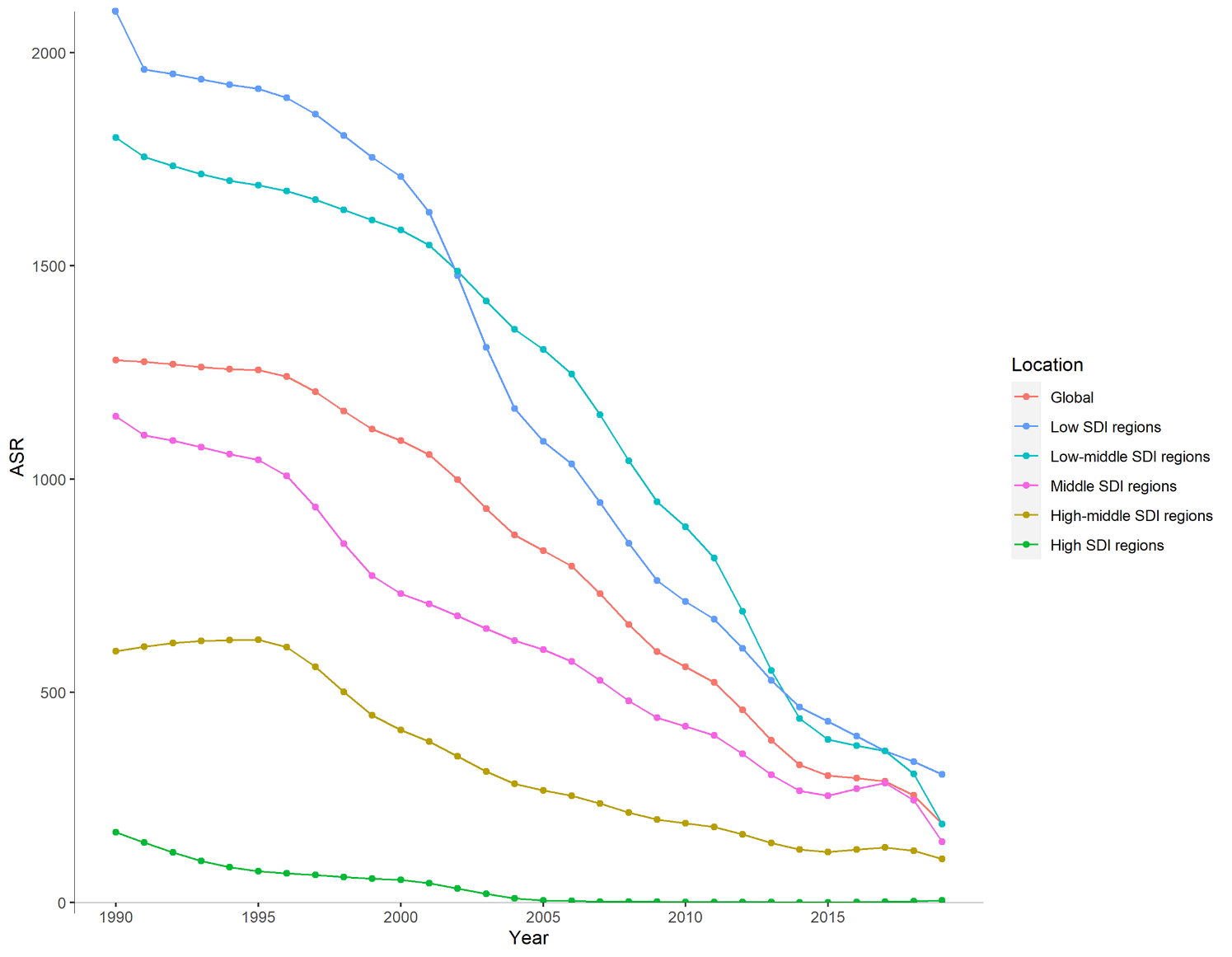


**Supplementary Figure 2.** The trend of ASR of measles by region from 1990 to 2019. SDI: socio-demographic index


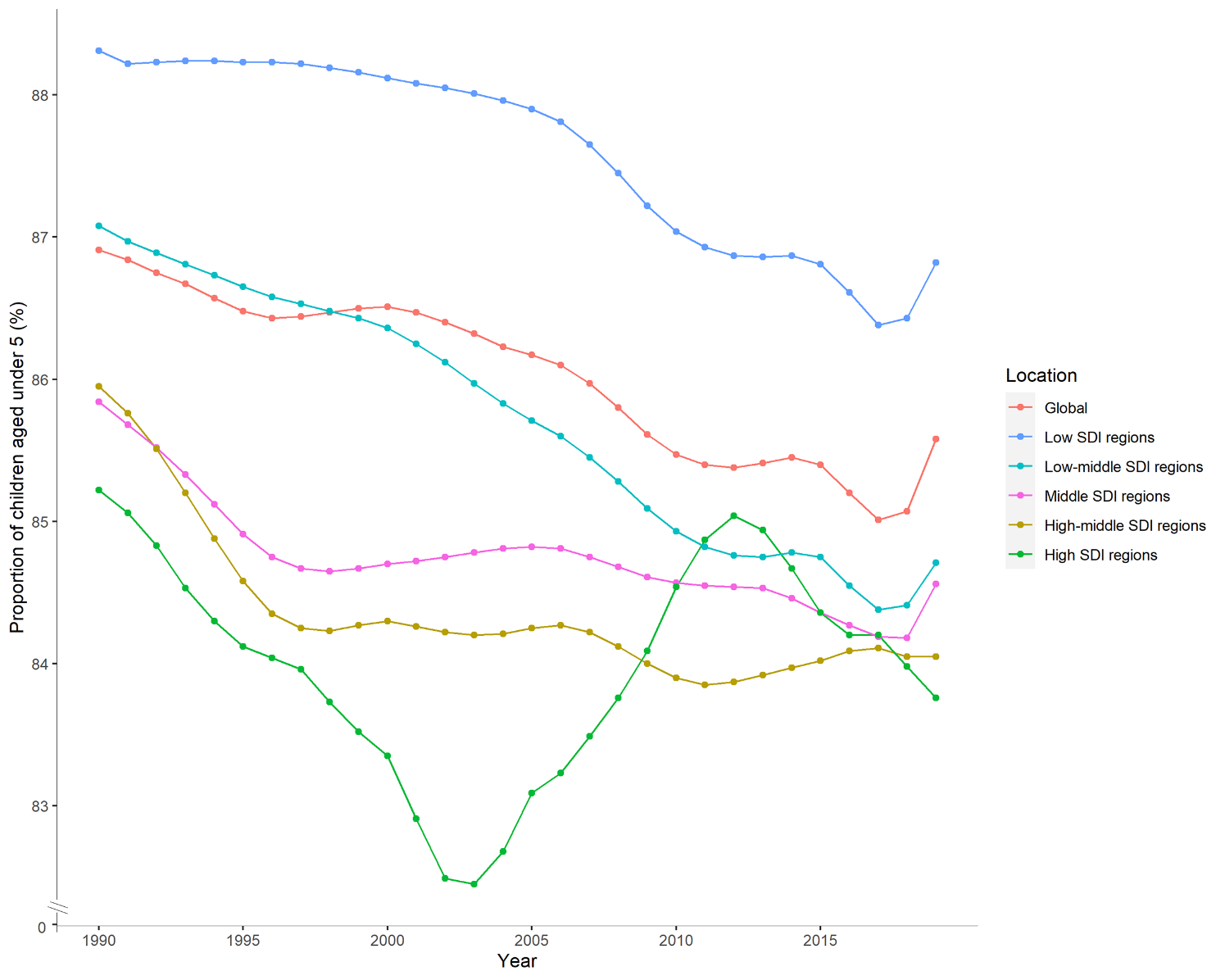


**Supplementary Figure 3.** The proportion of children aged under 5 among total measles incidence cases by region from 1990 to 2019


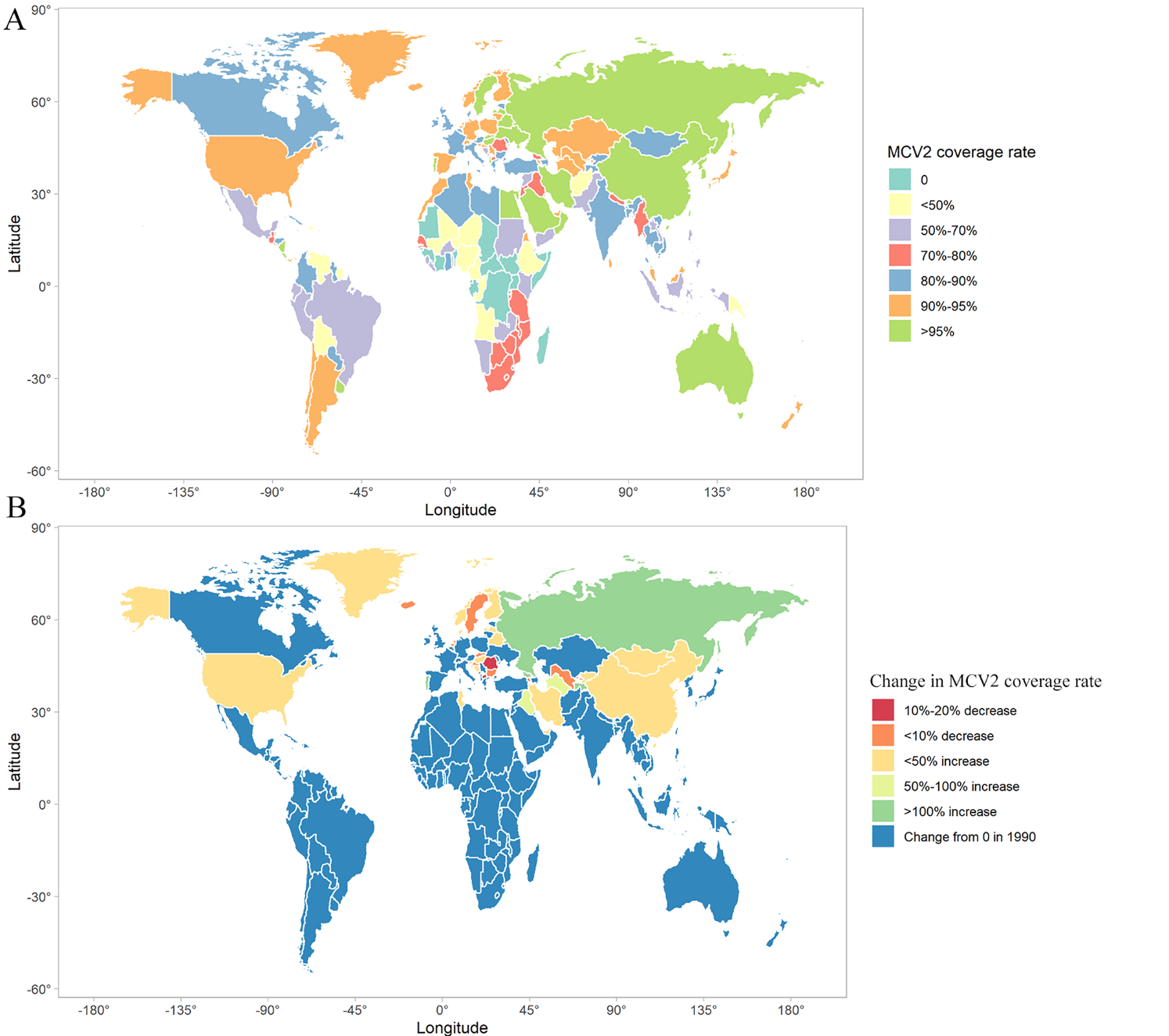


**Supplementary Figure 4.** The global trends in MCV2 coverage in 204 countries and territories. (A) MCV2 coverage rate in 2019; (B) changes in MCV2 coverage rate between 1990 and 2019. MCV2: measles-containing vaccine, dose 2


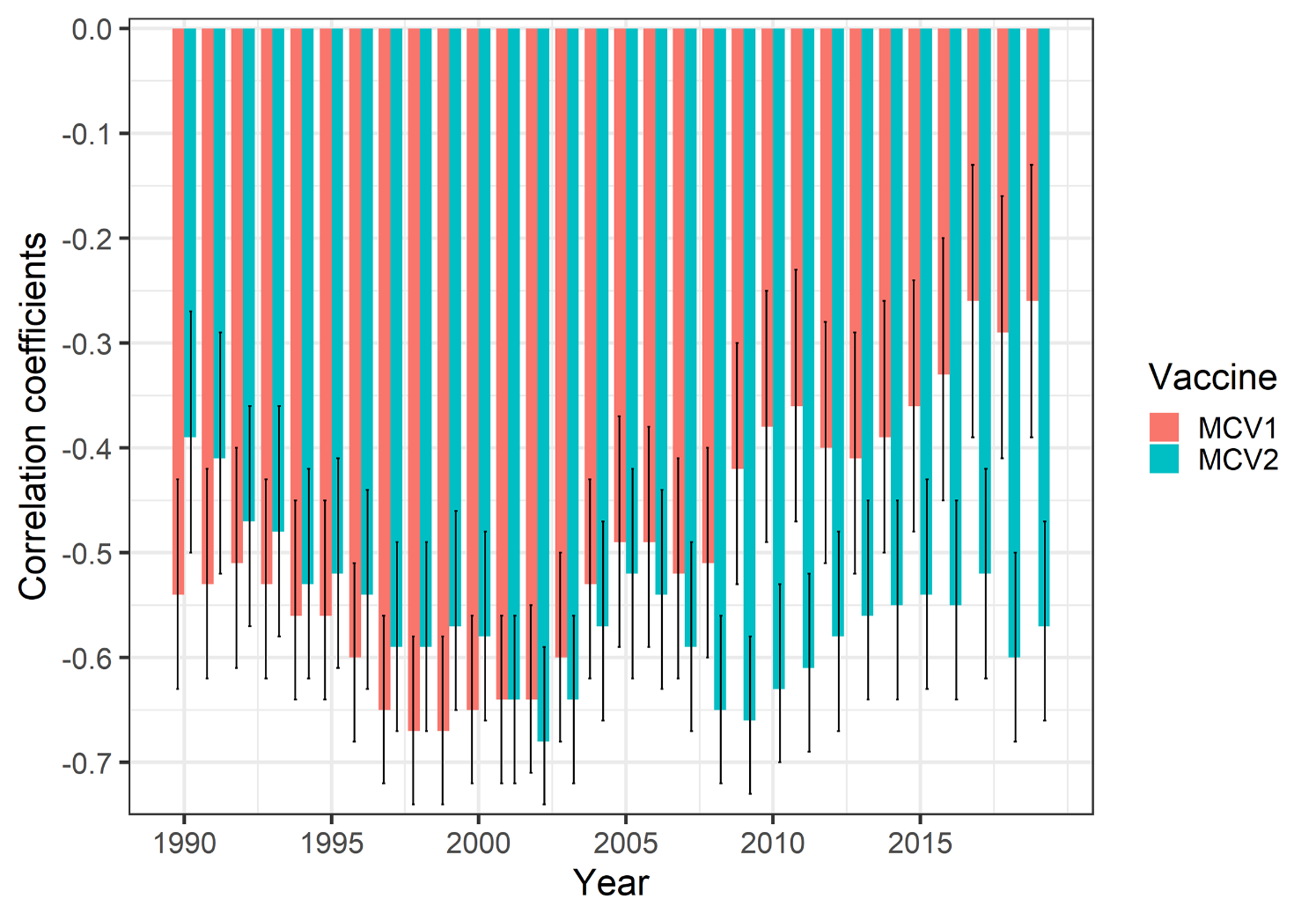


**Supplementary Figure 5.** The correlation between ASR and MCV1 and MCV2 coverage rate from 1990 to 2019 at national level. MCV1: measles-containing vaccine, dose 1; MCV2: measles-containing vaccine, dose 2; ASR: age-standardized rate.
